# Codesign and refinement of an optimised antenatal education session to better inform women and prepare them for labour and birth

**DOI:** 10.1136/bmjoq-2023-002731

**Published:** 2024-06-10

**Authors:** Abi Merriel, Miriam Toolan, Mary Lynch, Gemma Clayton, Andrew Demetri, Lucy Willis, Narendra Mampitiya, Alice Clarke, Katherine Birchenall, Chloe de Souza, Emma Harvey, Tamarind Russell-Webster, Eva Larkai, Mariusz Grzeda, Kate Rawling, Sonia Barnfield, Margaret Smith, Rachel Plachcinski, Christy Burden, Abigail Fraser, Michael Larkin, Anna Davies

**Affiliations:** 1 Department of Women's and Children's Health, University of Liverpool, Liverpool, UK; 2 Department of Women's and Children's Health, North Bristol NHS Trust, Bristol, UK; 3 Academic Women's Health Unit, University of Bristol, Bristol, UK; 4 Population Health Sciences, University of Bristol, Bristol, UK; 5 University of Bristol, Bristol, UK; 6 North Bristol NHS Trust, Westbury on Trym, Bristol, UK; 7 King's College London, London, UK; 8 Parent Representative, Bristol, UK; 9 NCT User Representative, London, UK; 10 Institute of Health and Neurodevelopment, Aston University, Birmingham, UK

**Keywords:** Maternal Health Services, Obstetrics and gynecology, Healthcare quality improvement, Patient education

## Abstract

**Objective:**

Our objective was to codesign, implement, evaluate acceptability and refine an optimised antenatal education session to improve birth preparedness.

**Design:**

There were four distinct phases: codesign (focus groups and codesign workshops with parents and staff); implementation of intervention; evaluation (interviews, questionnaires, structured feedback forms) and systematic refinement.

**Setting:**

The study was set in a single maternity unit with approximately 5500 births annually.

**Participants:**

Postnatal and antenatal women/birthing people and birth partners were invited to participate in the intervention, and midwives were invited to deliver it. Both groups participated in feedback.

**Outcome measures:**

We report on whether the optimised session is deliverable, acceptable, meets the needs of women/birthing people and partners, and explain how the intervention was refined with input from parents, clinicians and researchers.

**Results:**

The codesign was undertaken by 35 women, partners and clinicians. Five midwives were trained and delivered 19 antenatal education (ACE) sessions to 142 women and 94 partners. 121 women and 33 birth partners completed the feedback questionnaire. Women/birthing people (79%) and birth partners (82%) felt more prepared after the class with most participants finding the content very helpful or helpful. Women/birthing people perceived classes were more useful and engaging than their partners. Interviews with 21 parents, a midwife focus group and a structured feedback form resulted in 38 recommended changes: 22 by parents, 5 by midwives and 11 by both. Suggested changes have been incorporated in the training resources to achieve an optimised intervention.

**Conclusions:**

Engaging stakeholders (women and staff) in codesigning an evidence-informed curriculum resulted in an antenatal class designed to improve preparedness for birth, including assisted birth, that is acceptable to women and their birthing partners, and has been refined to address feedback and is deliverable within National Health Service resource constraints. A nationally mandated antenatal education curriculum is needed to ensure parents receive high-quality antenatal education that targets birth preparedness.

**WHAT IS ALREADY KNOWN ON THIS TOPIC**

- Antenatal education is used to prepare women/birthing people for labour, birth and the postnatal period, but it has been eroded. Antenatal education has potential to support women/birthing people in developing their expectations around labour and the postnatal period, via improved health literacy. Improving antenatal education could be impactful as the expectation–experience gap is linked to post-traumatic stress disorder.

**WHAT THIS STUDY ADDS**

- We have shown that a codesigned, optimised antenatal class can provide information helpful to parents and important to staff, within the constraints of the National Health Service resources.

**HOW THIS STUDY MIGHT AFFECT RESEARCH, PRACTICE OR POLICY**

- This study can be used to understand what parents need from antenatal education, and how to begin to address the expectation–experience gap.

## Introduction

Antenatal education (ANE) has been used to prepare women/pregnant people for labour and birth for many years.^1^ It is a vital element of antenatal care and is incorporated into The National Institute of Health and Care Excellence (NICE) guidelines.^2^ ANE contributes to practical preparation, but it can contribute to a woman/pregnant person’s expectations and experience of labour and birth and consequently their psychosocial outcomes.^3^

When considering what is important to them about birth, women prioritise the physiological birth of a healthy baby. However, when things do not go according to their plan, they wish to retain a sense of personal achievement and control through active decision-making.^4^ Empowering women/pregnant people to participate in this process through high-quality ANE has been shown to mediate childbirth satisfaction.^5^

Antenatal preparation has the potential to support women/pregnant people in developing their expectations. This is important because an expectation–experience gap increases risk of post-traumatic stress disorder (PTSD).^5^ The triggering event is likely to be varied, from not receiving a caesarean birth when the preference was for one, through to negative childbirth experiences.^5^ The origins of a woman/pregnant person’s PTSD do not lie solely with birth experiences, although intervention, pain and a negative perception of labour care are risk factors for its development. Coexisting factors, for example, higher trait anxiety and antenatal depression scores also contribute to its development.^5 6^ High rates of stress-related symptoms are experienced following unanticipated intervention in labour. Up to half have PTSD 2 months after unplanned caesarean compared with 24% at 6 weeks after vaginal birth.^7^ Risk factors for PTSD include subjective birth experience relating to negative emotions and lack of control or agency, operative birth and lack of support from staff during birth.^6^ Up to 1.5% of women experience PTSD 6 months postnatally.^8^ There may also be a link between birth expectations and depression.^5^ Good-quality ANE provides an understanding of common interventions that might become necessary, and could attenuate the expectation–experience gap.

ANE provision is variable, less than a third of women are offered antenatal classes.^9^ ANE is available within the National Health Service (NHS), privately for profit or not for profit, by clinicians and allied healthcare staff, or by trained antenatal educators. ANE can be traditional information provision classes or focused on self-directed coping strategies, for example, hypnobirthing; some pregnancy exercise classes also provide elements of education and preparation. Different classes may have a particular focus (eg, physiological birth) or a clear goal to provide evidence-based information. This area is unregulated and although NICE recommends ANE for all women in their first pregnancy, they do not provide comprehensive guidelines on what should be covered in ANE classes.^2^

Prior to this intervention development study, we conducted focus groups with 48 postnatal women.^10 11^ They highlighted their experiences of the gap between expectations and outcomes, the impact of this on their well-being and the need to improve the quality of ANE. They described limited discussion of common interventions (eg, assisted birth, induction) and birth experiences (eg, perineal trauma) resulting in them believing that these events were infrequent and unnecessary to learn about. Participants reflected that receiving sensitively provided information about the frequency and nature of interventions and common events during birth was important and could support psychological health if birth experiences were not as expected. When discussing ANE with midwives (unpublished—online supplemental file 1), we found that many were not provided with specific training to deliver ANE, nor did they enjoy or want to deliver it. Furthermore, midwives often designed the class materials themselves, with little assistance or guidance.

10.1136/bmjoq-2023-002731.supp1 Supplementary data

We aimed to codesign, implement, evaluate acceptability and refine an optimised ANE session to improve birth preparedness for future implementation. This was part of the Antenatal Care and Education project and so named the ‘ACE’ intervention.

## Methods

The methods are presented in the four phases: codesign (2019), trial implementation (2021–2022), evaluation and refinement (2021–2022).

### Research team and organisational commitment

The initial research team was made up of obstetricians (AM, MT, SB and CB), midwives (MLynch and MS), psychologists (ADavies, EA and MLarkin), service users (KR), NCT representative (RP), epidemiologist (AF), project manager and medical students (TR-W and EL). As the project progressed and was impacted by COVID-19, additional trainee obstetricians (ADemetri, CdS, KB), doctor-in-training (AC) and medical students (LW, NM, EH) joined the research team.

ACE was supported by the Head of Community Midwifery (MS) and the then Head of Obstetrics (SB). This facilitated delivery of the study and will enable future roll out.

### Patient and public involvement

Parents were involved from the inception of the study and played an active role in the design. A parent sat on the study steering group (KR) and parents codesigned the ACE intervention, drawing on the data generated in focus groups/survey studies.

### Study setting and context

This study was delivered in a single hospital in the South-West of England with approximately 5500-6000 births annually. It was conducted before and during the COVID-19 pandemic (2019–2022) when there was limited provision of ANE and limited contact between staff and women.^12^

### Frameworks to inform intervention development

We used the Medical Research Council’s Complex Intervention Development Framework (2008) to develop the ACE intervention.^13^ This allowed us to consider the key stages of intervention development and refinement to inform robust development of ACE (development, feasibility, evaluation and implementation). The focus of this study is on the development and some feasibility aspects of this framework. However, it is useful to consider the whole picture when planning research and therefore the evaluation. The consolidated framework for advancing implementation science was used to plan and support initial testing and further development of the intervention,^14^ to ensure preparedness for implementation. By focusing on intervention characteristics, outer setting, inner setting, characteristics of the individuals involved and the process of implementation, we were able to ensure that these areas were addressed to facilitate a robust approach to development, design and initial evaluation of the ACE session.

### Phase 1: co-design

**Figure 1:**
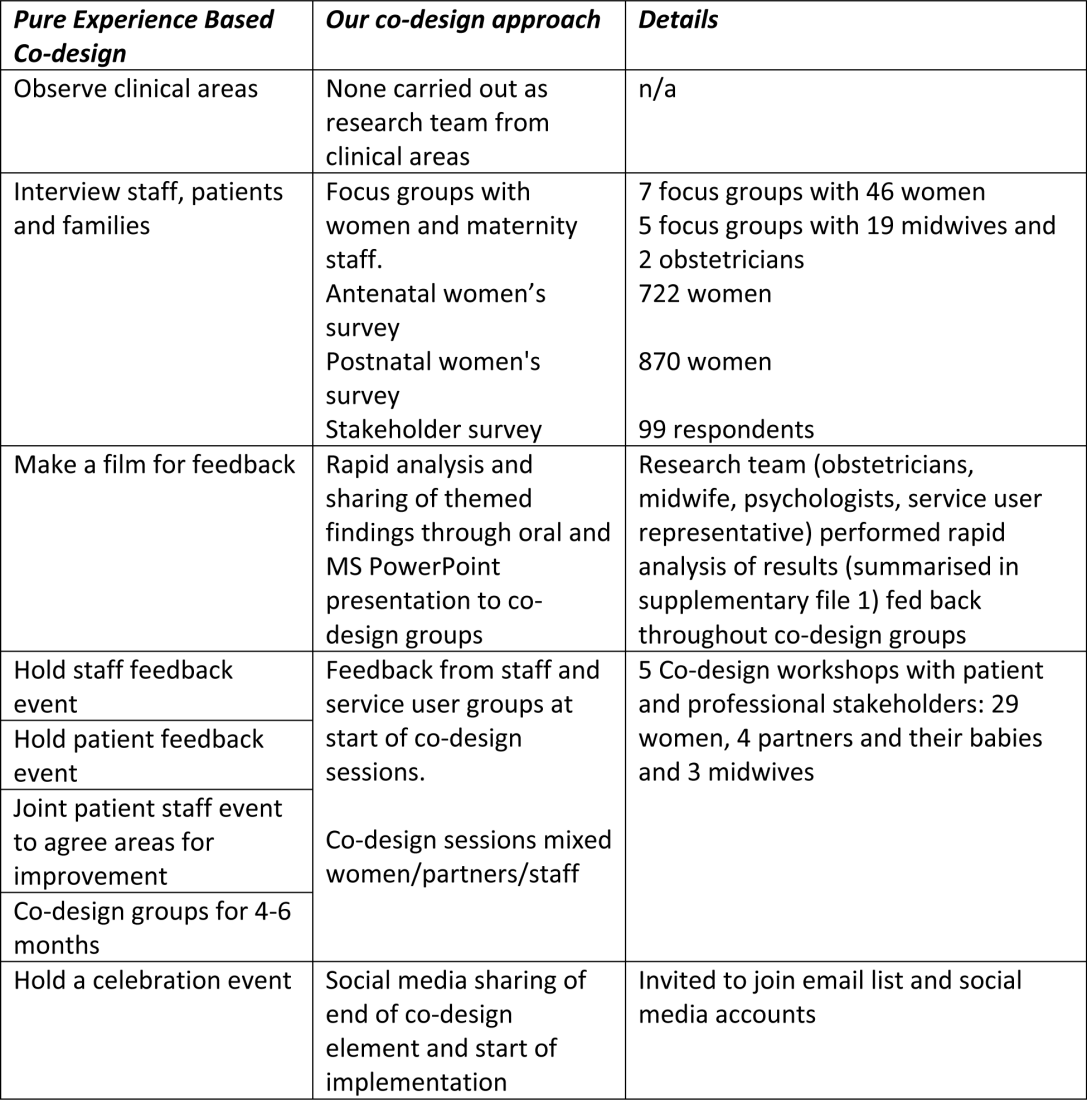
Experienced-Based Co-Design steps and our adaptions

Co-design was chosen as a method to involve service users in the development of the ACE session because patient experience, outcomes and safety are linked.^15^ We saw the best way to improve patient experience as being to involve service users in the intervention design. We used an adapted experience-based co-design (EBCD) approach.^16 17^ Figure 1 shows the EBCD steps and our adaptations to them. We included the two core co-design elements: service user experience data and including service users in the design.^17^ The planned output from the codesign was a 2-hour ACE session on labour and birth, and materials to deliver the session.

### Phase 2: implementation

#### Recruitment of antenatal educators

We recruited community midwives who deliver ANE. Those agreeing to take part were invited to a training session. All time contributed to training and delivery of ACE was remunerated via the midwifery ‘bank’ system.

#### ANE group attendees

We invited women/birthing people who were over 18 years of age, more than 24 weeks pregnant and in the care of the local trust to participate. We informed them that the ACE session was a supplement to their NHS-offered session. They were asked to invite their partners if they wished. Recruitment was via (1) social media, (2) community midwifery referrals and (3) telephone calls to all eligible pregnant women/birthing people. Women/birthing people and their partners were given written information via email. They then booked into a session on the date of their choice, completed an online consent form and provided basic demographic information.

#### Impact of the COVID-19 pandemic

We paused this study as face-to-face ANE was halted soon after the co-design groups had finished. We restarted this study when face-to-face interactions were permitted and ran ACE classes in a COVID-secure way. Parents were therefore invited to a central location in the evenings, rather than the originally planned local venues.

#### Training in delivering the ACE session

A training session was held for midwives. As provision expanded, midwives new to the programme attended an ACE session delivered by an experienced facilitator and discussed it with her.

#### Cost of implementation

Reusable resources included a projector, printed posters and attachable reusable stickers, costing £150–200. Each woman/birthing person requires a printout of the ‘what is important to me’ tool. Existing resources (eg, pelvis and doll, kiwi cups) were used. After midwifery training, the remainder of the costs were identical to existing costs to deliver ANE.

### Delivery

The session was 2 hours long and (due to COVID-19) restricted to 10 participants and their partners. The research team observed each session, noting attendance and contemporaneously recording the extent to which the intervention was delivered as intended (fidelity), and the session length. Women received a £10 voucher for their expenses.

### Phase 3: evaluation

The ACE class was evaluated/refined in five ways:

1. Immediate debrief with midwife delivering the session
  This discussion between the midwife and research team was recorded on a structured form to identify successes and areas for improvement. The structured feedback form was designed to align with the core components of the class manual. Feedback was reviewed after each session by the study lead (AM)/trial manager (ADavies) to identify any immediate changes to implement.
2. Online questionnaire for women/birthing people and birth partners (BPs).
  An online survey hosted in REDCap^18^ was sent out within 2 weeks of attending the session. The questionnaires used for collection of parent feedback were designed by the multidisciplinary research team to address the feedback required, and piloted by team members including parents. Participants were asked to rate the content, delivery and resources and how useful the session was in preparing them for childbirth. They were asked if they had attended other ANE, and if so to compare the ACE sessions to these. Quantitative data were analysed in STATA, and themes from free-text feedback were coded by a researcher. This was analysed at the end of the study to provide overall feedback and identify additional refinements.
3. Semi-structured feedback interviews with women/birthing people±BP approximately 2 weeks after attending the ACE session and/or 4–6 weeks after birth. The semi-structured interview topic guides were designed by the multidisciplinary research team and used flexibly to elicit information about the participants experience of the class.
  At the end of the classes women/birthing people±their BPs were asked to express interest in being interviewed about the class. Interviews were conducted either before their baby was born (approximately 2 weeks post ACE session), or 4–6 weeks after their baby was born. Parents could take part in one or both interviews. These could be conducted alone or with their partner in attendance. We purposively sampled parents who had attended sessions delivered by different midwives, both at the start and end the implementation capture a range of experiences.
  Interested parents were offered written information and a mutually convenient time for a telephone interview. Informed consent was obtained via a form hosted in REDCap.^18^ The interviews were audio recorded and rapid thematic analysis performed directly from the audio recordings to identify areas for change and areas where feedback was positive. A £10 voucher was provided.
4. Midwife focus group
  We undertook a single 1-hour focus group with the midwives who delivered the ACE class to gather feedback about their experiences of the class, the manual and any required improvements. The topic guide centred around these areas and was developed by the multidisciplinary research team. The group was audio recorded and rapid thematic analysis carried out from the audio files. A £10 voucher was provided.

### Phase 4: refinement and reporting of findings

The findings from the immediate session feedback were iteratively implemented into the intervention. The findings from the online questionnaires and from the focus groups were analysed once at the end of the study and therefore refinements were only included in the updated ACE resources (online supplemental file 2).

10.1136/bmjoq-2023-002731.supp2 Supplementary data

Potential changes to the manual and materials were recorded in an adapted table of changes.^19^ This shows the changes suggested, how frequently, whether it was feasible and reasonable to make the change and what change was actually made. This provided a systematic, rigorous approach to identifying potential changes, facilitating discussions between the study team, and agreeing final changes.

## Results

### Phase 1: codesign

Five codesign groups were undertaken with 29 women, 4 partners and 3 maternity staff. They developed the concept for the ACE class, the topics, planned the materials and discussed the importance of training for the staff.

The group designed the ACE class around a river journey, conceptualising the process of birth as a journey down the river, with a winding course that represented the different stages of labour that may be experienced before reaching birth. The river served as a metaphor to illustrate that labour and birth could take a number of different courses, but all would end in a postnatal bay with their baby, where support from friends, family and healthcare professionals would be available.

The topics identified in response to the focus group data were as follows: differing birth journeys (spontaneous vaginal, induction, assisted vaginal birth, planned caesarean, unplanned caesarean); coping with labour and birth (pharmacological and non-pharmacological); the immediate postnatal period; birth preferences; and social support. The codesign group believed a variety of different birth experiences should be reflected within the ACE session. We therefore made videos of their experiences of vaginal births, assisted vaginal births, quick and long inductions of labour and planned and emergency caesarean births.

The codesign group planned a ‘what is important to me’ birth preferences tool, to support attendees in considering their birth preferences. The tool focuses on the birth of a healthy baby at the end of labour and how their preferences could aim to achieve this (online supplemental file 2).

### Phase 2: implementation

A 3-hour training session for midwives was designed and delivered online and in person as desired. The ACE manual provided a session outline. Five midwives ran 19 ACE sessions, delivered to 142 women and 94 partners. On two occasions, midwives were unable to attend within 6–12 hours of the session; the project lead (AM—obstetrician) delivered the sessions to avoid inconvenience. The demographics of those attending the session are presented in table 1.

**Table 1:** Demographics of women and birth partners attending the sessions and completing feedback

	All session attendees				Attendees completing feedback			
	Mothers (n=143)		Partners (n=94)		Mothers (n=121)		Fathers (n=33)	
Age (years)								
(Mean, SD)	31	4	34	7	31	4	34	5
Education								
Asian or Asian British	6/143	4%	5/94	5%	3/118	3%	1/32	3%
Black or Black British	7/143	5%	1/94	1%	5/118	4%	1/32	3%
Chinese	0/143	0%	1/94	1%	0/118	0%	1/32	3%
Mixed ethnic background	1/143	1%	4/94	4%	1/118	1%	27/32	84%
White	127/143	89%	80/94	85%	107/118	91%	1/32	3%
Other ethnic group	2/143	1%	2/94	2%	2/118	2%	1/32	3%
Not known			1/94	1%	9/118	8%	4/31	13%
Education								
GCSE*/equivalent	11/142	8%	13/92	14%	13/118	11%	4/31	13%
A-levels/equivalent	20/142	14%	10/92	11%	52/118	44%	11/31	35%
Bachelors degree/equivalent	61/142	43%	34/92	37%	34/118	29%	9/31	29%
Postgraduate degree	38/142	27%	25/92	27%	8/118	7%	1/31	3%
Other	10/142	7%	4/92	4%	2/118	2%	2/31	6%
Prefer not to say	11/142	8%	6/92	7%	34	3		
Gestational age (weeks)								
(Mean, SD)	34	3			118/118	100%		
No. of babies before this pregnancy								
0	140/143	98%			108/118	92%		
1	3/143	2%			7/118	6%		
No. of pregnancies (including this one)								
1	130/142	92%			2/118	2%		
2	9/142	6%			1/118	1%		
3	2/142	1%			15/118	13%	3/32	9%
8	1/142	1%			103/118	87%	21/32	66%
Attended NHS class in this pregnancy								
Yes	17/143	12%	10/94	11%			8/32	25%
No	126/143	88%	57/94	61%	59/118	50%	13/31	42%
No these were not available			27/94	29%	0/118	0%	1/31	3%
Attended non NHS class in this pregnancy								
Yes	66/143	46%	37/92	40%	16/118	14%	12/31	39%
Attended NHS class in previous pregnancy								
Yes	1/143	1%	3/92	3%	102/118	86%	18/31	58%
No	17/143	12%	34/92	37%				
Not been pregnant before	125/143	87%	55/92	60%				

GCSE, General Certificate of Secondary Education; NHS, National Health Service.

### Phase 3: evaluation

#### Fidelity of delivery

During the 19 sessions, most of the class content was covered consistently; in all or a majority of sessions, the following content was addressed or partially addressed: birth journeys (n=19/19), coping strategies (n=19/19), social support (n=14/19) and partner support (n=18/19). A social opportunity was provided inconsistently (n=8/19). Across groups, materials and resources were well used with the exception of the ‘what is important to me’ birth preferences tool which was not used in over half of the classes (n=8), although it was explained in the majority (n=17/19). Online supplemental file 3 provides detailed observations and feedback.

10.1136/bmjoq-2023-002731.supp3 Supplementary data

### Feedback from participants

#### Interviews

21 interviews were conducted: 13 antenatally and 7 postnatally. One couple completed both antenatal and postnatal interviews. For three interviews, women were interviewed with their partners. Participants valued attending a class with an NHS professional, feeling that it was an opportunity to get good-quality information about their local setting and the care they could expect. Most reported that the river concept was useful and that while a great deal of information was being given, it met their needs and was not overwhelming. Several women felt that more information around the impact of decisions, for example, induction of labour would be beneficial. They desired more opportunity for social interaction between class participants. Online supplemental file 4 contains a summary of themes and supporting quotes.

10.1136/bmjoq-2023-002731.supp4 Supplementary data

#### Questionnaires

121 women (W) and 33 BPs completed the online feedback form following the class (demographics in table 1). Most participants attended in their third trimester, were aged 30–35, were of white ethnicity (89% W, 85% BPs) and were university educated (70% W, 64% BPs).

**Figure 2:**
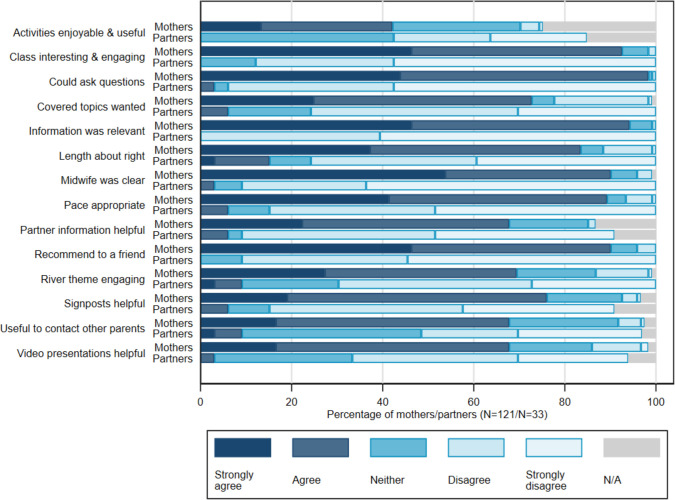
Feedback on Practical Elements of the ACE Class

**Figure 3:**
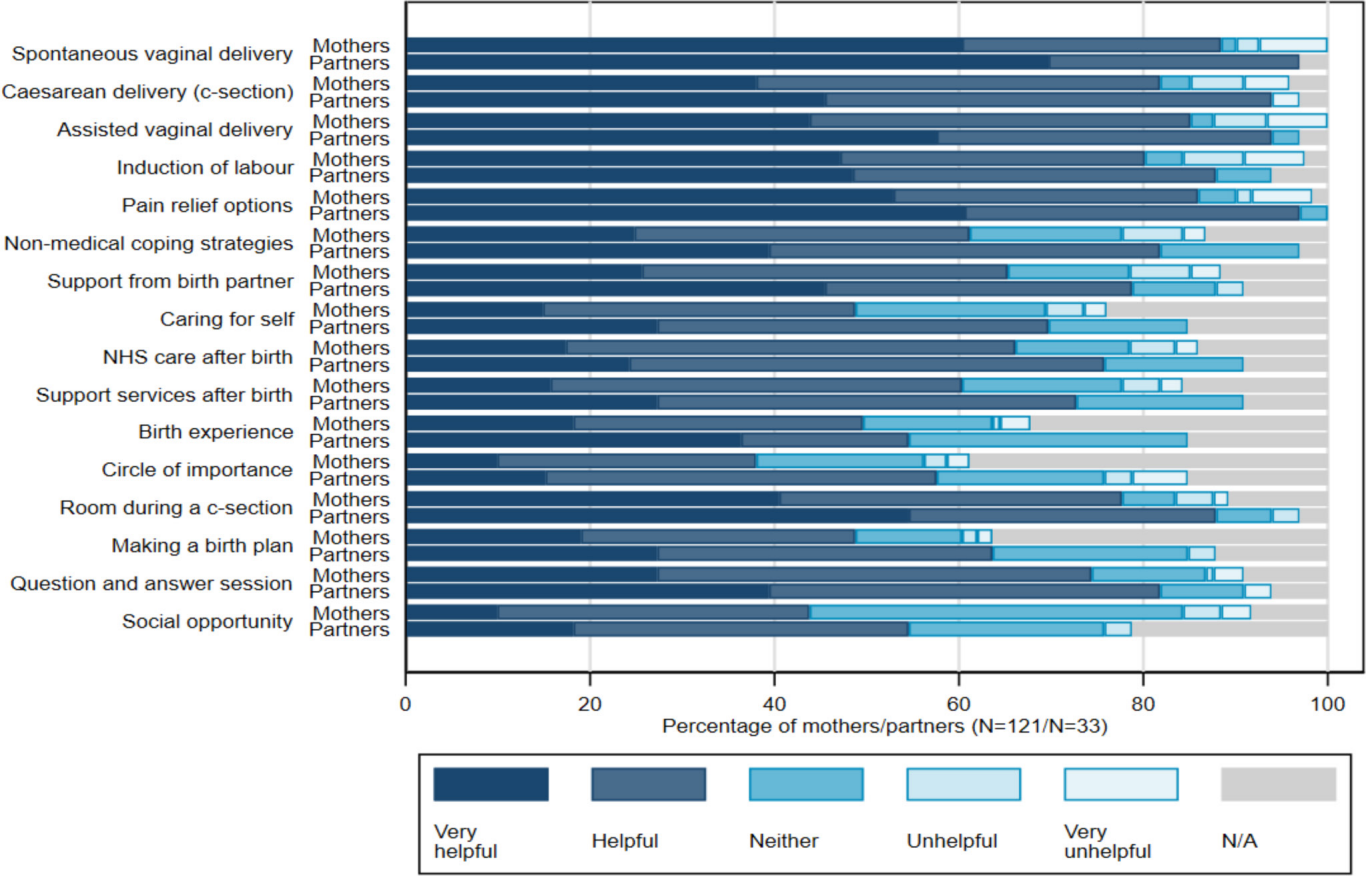
Feedback on Content of the ACE Class

When considering how prepared the participants felt after the class, 79% of women and 82% of BPs felt more prepared than they were beforehand. The majority reported having improved knowledge of strategies to cope with pain (W 79%, BP 82%) and to use if things did not go according to plan (W 81%, BP 85%). Further feedback is provided in figure 2. Women found the sessions more useful and engaging than their BPs. However, when considering the specific areas of information provided (figure 3), most women and their partners found much of the session content to be very helpful or helpful.

121 women responded to questions about the attendance of the BP, of these 90 (74%) women reported that their BPs attended the class. Of those whose partners did not attend, 21 reported that they could not make the session. Seventy-six per cent of women felt that it was very important that they could bring their partners to the class, and 95% of those whose partners attended thought that the class was useful to their BP. Most women (93%) stated that they would recommend to a friend that they bring a BP with them.

When comparing ACE with other classes, 58% of women and 52% of BPs planned to attend other classes, with the most popular being hypnobirthing (37% women, 24% BPs). When comparing ACE and NHS classes, only 11 women and 5 partners had attended NHS classes prior to their ACE session, preventing meaningful conclusions from being drawn. However, participants indicated that they received similar information and that both were useful. However, if they had to pick one class, the majority of participants stated that they would select the ACE session (W=64%, BPs=60%). After the class, there was an increase in the number of women who indicated that they had thought about strategies for coping with labour (91% post vs 78% pre) and knew how to find further answers to questions about labour and birth (89% post vs 64% pre).

#### Feedback from midwives

Midwives generally felt the session went to plan, liked the COVID-imposed small group sizes and the structure. However, they wanted more interaction and time to deliver the class. These data are presented in online supplemental file 5.

10.1136/bmjoq-2023-002731.supp5 Supplementary data

### Phase 4: refinement

#### During the intervention

Changes were made in response to the midwife focus group and the feedback from the interviews with parents. These included adding in more explanation of emergencies, rearranging the seating plan and the way the resources were displayed.

#### Postintervention refinement

We made changes where there were consistent reports from midwives, parents or both that a change should be made. These are displayed in table 2. Overall, 38 changes were recommended: 22 by parents, 5 by midwives, and 11 by both. We have incorporated 36 of these within the training manual (online supplemental file 2). We were unable to address two consistently requested changes: (1) the introduction of a postnatal session, because this was outside the remit of this project, and (2) improvement to the ethnic and socioeconomic diversity of the videos, as we were limited by those who were willing to be filmed. We did not have sufficient funding to develop further videos.

**Table 2:** Table of changes made to intervention following feedback

Recommendation	By	Solution	Action
Content			
Guidance on writing birth preferences	P	Discuss birth preferences tool supported by a poster including information about local and national birth preference tools.	Rewrite section in manual
More information on the support partners can offer and their role	P	Reiterate to midwives at start of manual how important this is	Emphasise in manual
More information on what affects advice on place of birth	P	Short section in manual highlighting it to midwives	Addition to manual
Include what to put in hospital bag	P	Interactive ‘hospital bag’ exercise, discuss contents more formally, highlight NHS tool	Addition to manual
Include lay out of hospital	P	Suggest direction to local website/tours	Addition to manual
Outline what equipment available and how to ask for it	P	Incorporate discussion into coping strategies section	Addition to manual
More information on pain management not just drugs	P	Highlight coping strategies to midwives	Emphasise in manual
More information on induction and impact on labour and birth	P	Incorporate impact into discussion of induction of labour	Addition to manual
Teach hands on massage	P	Not suitable to incorporate into class as not all women attend with a partner	No change
Practical teaching and discussion on breathing techniques	P	Incorporate into coping strategies element	Addition to manual
Enact labour positions	P	Could be culturally inappropriate for some parents/awkward in available space	No change
Include more on theatre/emergencies	M	Incorporated already during implementation, add section to manual	Addition to manual
Need more information on post birth recovery	P/M	Add some immediate postnatal information	Addition to manual
Cover newborn care/life with baby	P/M	Outside remit of this class direct to postnatal/infant feeding class	
Infant feeding methods	P	Outside remit of this class direct to postnatal/infant feeding class	
Structure			
Needs to be more interactive	P/M	Add in hospital bag exercise, add in breaks to chat, ensure time at end for people to discuss	Emphasise in manual
Need to be able to get up and move	P	Add it 2×5 min breaks	Addition to manual
Sitting in rows does not support interaction	P/M	Set up in circle if feasible or around a table.	Emphasise in manual
Need to have two sessions/more time	P/M	No change—not in remit	
Integrate coping strategies throughout	M	Emphasise to midwives to draw in coping strategies throughout	Emphasise in manual
Late in the evening	P	Offer a variety of times	Emphasise in manual
Presentation			
River concept not well integrated	P/M	Encourage staff to use concept and gain experience	Emphasise in manual
Use more empowering language	P	Emphasise to staff the importance of language	Emphasise in manual
Staff need to be consistently trained and confident with material	P	Develop the manual further and clear training	Training video and manual
Did not use or explain post it notes	P	Each midwife to choose personal mechanism for enabling parents to ask questions	Emphasise in manual
Two people presenting would be better	M	Recommendation in manual that if two people are able to facilitate the session it would be easier to deliver session	Emphasise in the manual
Resources			
Couldn’t see the labour posters well	P/M	Local trusts to use their existing posters/resources to support the explanation of labour	Use existing resources
Posters fell off the wall	P/M	We developed a solution in the manual, provide our solution in the manual	Addition to manual
Use all first time parents in videos	P	Classes are not restricted to first time parents only. Could raise additional funds to make extra videos	Future action
Use more diverse parents in videos	P	We acknowledge this issue, however, were only able to make videos with those who volunteered.	Future action
Ensure video’s work	P/M	Ensure staff are able to work laptops and projector	Emphasise in manual
Could share link to videos	P	We are unable to do this as do not have permission to use them outside of the class but acknowledge this is a good idea for future.	Future action
Need a picture for waters breaking	M	Develop additional picture.	Add to manual
Show forceps in the class	P/M	Encourage midwives to borrow forceps for the class	Emphasise in manual
Birth preferences tool not useful, but concept of considering what is most important to you is.	M	Emphasise the discussion of the concept of considering what is important but remove the idea of printing the tool	Alteration to manual
Other			
Feeling that the content was medicalised	P/M	Ensure that time is given to non-pharmacological coping strategies and that there are no expectations of interventions; however, due to the codesign process and other positive feedback, we are not planning to remove discussion about interventions.	Emphasise in manual
A lot to remember, needed to take notes	P	Encourage signposting to local/national resources	Add a manual section
Conflicting information with other providers, for example, pethidine being bad versus good	P	Reminder that that the information in the manual is as far as is possible inline with National Institute for Health and Care Excellence guidelines and that this class, when delivered as codesigned is evidence based and balanced	Emphasise in manual

NHS, National Health Service.

## Discussion

In this paper, we present the co-design and feasibility work of an optimised 2-hour ANE session for labour and birth. The ACE birth journeys ‘river’ concept was developed by the co-design groups and delivered by local clinical staff to participants. Through implementation in an NHS setting and evaluation with both participants and providers, we systematically identified key issues in the delivery of the session both concurrently and after completion of the classes. We used a collaborative, systematic decision-making approach to addressing issues that were consistently reported by women, midwives or both leading to refinements, to the ACE manual, and the development of a session that can be delivered within the constraints of NHS capacity.

In general, women/pregnant people reported enjoying the session, and found it informative and useful in helping them to prepare for birth. However, it is notable that partners described being less engaged with the class. Nonetheless, both women/pregnant people and their partners who provided feedback found specific content of the session useful. Women/pregnant people reported increased preparedness, having thought more about coping strategies for labour, and knew how to find further information following the session compared with before attendance.

In this study, we have explored potential impacts of the ACE intervention on women/pregnant person’s preparedness and informedness. This contributes to the data on ANE interventions, which are heterogenous. They include studies on general ANE,^20–22^ promotion of self-efficacy^23^ or mindfulness,^24^ with some focussing on specific topics or outcomes such as preventing postnatal depression,^25^ coping with fear^26^ and breast feeding.^27 28^ Many of these interventions have been tested in the context of a clinical trial and their length varies from a few hours to multiple sessions over several weeks. These interventions have not been tested within the NHS. Furthermore, given current NHS resource constraints, they are unlikely to be feasible to deliver in the context of current care.

There are some key differences between our approach and that of other studies to evaluating the impacts of the ACE intervention in terms of plausible effects of ANE. Many studies into ANE focus on clinical outcomes, for example, rates of epidural and mode of birth.^29^ We would question whether it is the role of ANE to alter mode of birth, and challenge whether value should be placed on reducing or increasing epidural rates—women/pregnant people should be able to select their personal preference.

Our focus was to give information to support preparedness and informed decision-making. We believe it may be more beneficial for evaluations of ANE to examine outcomes related to what ANE should aim to achieve, for example, feelings of preparedness for birth, knowledge of the process of labour and birth, birth expectations and whether their birth has met them, and a sense of empowerment. Elements of existing systematic reviews do suggest that ANE can positively impact the labour and birth journey by reducing false labour admissions, which can be stressful for the mother, reduce anxiety and increase partner involvement.^30^ A more recent review^31^ has suggested that ANE can impact maternal stress and improve self-efficacy.^31^ A review focusing on childbirth self-efficacy alone suggested that ANE promotes women’s self-belief and is effective in achieving a positive birth experience.^23^ Our study did not specifically measure these outcomes, as our aim was to develop and refine the intervention; however, it is plausible that this co-designed class, covering general birth preparation within the context of a 2-hour session, could contribute to improving experience of birth for women in resource-constrained environments such as the NHS, and should be the focus of future research.

Of particular importance in our findings was the feedback from BPs. While they found the information useful, they reported that the class did not meet their needs. This finding is in keeping with existing literature suggesting that BPs feel outnumbered, excluded, anxious and uncertain and require more targeted birth preparation.^32^ A recent large qualitative study has highlighted the importance of ANE in meeting the needs of BPs^33^; however, there is less literature on exactly how the existing classes could be modified, or new classes designed, to meet the needs of BPs. While we gathered data from a small number of BPs during the codesign stage and after evaluation to inform the changes made to the ACE intervention, a limited number of partners took part in them, meaning that it is unclear whether their views were representative. Further research is needed, and should specifically target BPs, to explore in greater detail their needs to further refine the ACE class.

### Strengths and limitations

An important strength of the ACE intervention is that it was delivered within the allotted 2 hours, by NHS staff and women/pregnant people felt that the intervention was acceptable and useful. This may make it more feasible to roll out across other NHS Trusts. A further strength is that where possible, we iteratively addressed issues throughout the implementation period, to improve the experience for participants, and we sought feedback from multiple sources to enable us to refine the class. Furthermore, this intervention is underpinned by the experiences of women and pregnant people who have recently given birth.

However, we acknowledge that focus groups undertaken lacked ethnic and socioeconomic diversity. We attempted to diversify the sample attending the class by contacting all women greater than 24 weeks of pregnancy booked at the trust on up to two occasions to increase engagement. Despite these efforts, we did not achieve representation of women/birthing people that was consistent with our local population. There is evidence of barriers to attendance at ANE in women from underserved groups^34^; therefore, further efforts to identify ways to better engage these groups are needed. This may be achieved through public–patient involvement activities to identify barriers and how to address them. Increasing accessibility by providing sessions in local settings may increase attendance and provision of classes in other languages could address potential barriers. A weakness identified by attendees was that the class focused solely on the labour and birth element of ANE; this was due to an existing class in our trust that specifically addresses infant feeding. Finally, COVID-19 restrictions limited the location of the class and the spacing of attendees within it. A physically larger venue was required which meant classes were held centrally. This may have reduced the opportunity for interaction and relationship forming between participants as they attended the class to suit them, rather than the one held in their locality.

A further limitation is that this study was designed as an intervention development study and not an efficacy study; therefore, we were unable to evaluate the downstream effects of the impact of the intervention on participants’ knowledge or behaviours, experience of birth and PTSD. This is a key area for future research, and we would recommend that this should be the focus of a future trial. Finally, while we have co-designed the ACE programme, it is not yet rolled out and adopted into local practice; therefore, a future quality improvement project will be required to facilitate the roll-out and sustainability of the ACE classes into NHS trusts, if it is proven to be efficacious in a future trial.

## Conclusion

We have co-designed a structured ANE session about labour and birth that provides women/pregnant people with the information they want and need to prepare for birth, within the constraints of available NHS resource. This intervention was positively received by parents. Therefore, the next step is to work towards all parents having access to these classes, to support their journey into birth and beyond. To achieve this, national ANE guidelines are urgently needed to ensure equitable access to ANE and appropriate resources made available to embed and evaluate ANE.

## Data Availability

Data are available upon reasonable request. Most data are presented in the text and supplemental material. The qualitative data are presented as quotes. Data will be available upon reasonable request and with appropriate ethical permissions.
