# Supplementary figures and images for: Codesign and refinement of an optimised antenatal education session to better inform women and prepare them for labour and birth
Source: BMJ Open Qual. 2024 Jun 10;13(2):e002731. doi: 10.1136/bmjoq-2023-002731 (PMC11168157; doi:10.1136/bmjoq-2023-002731)

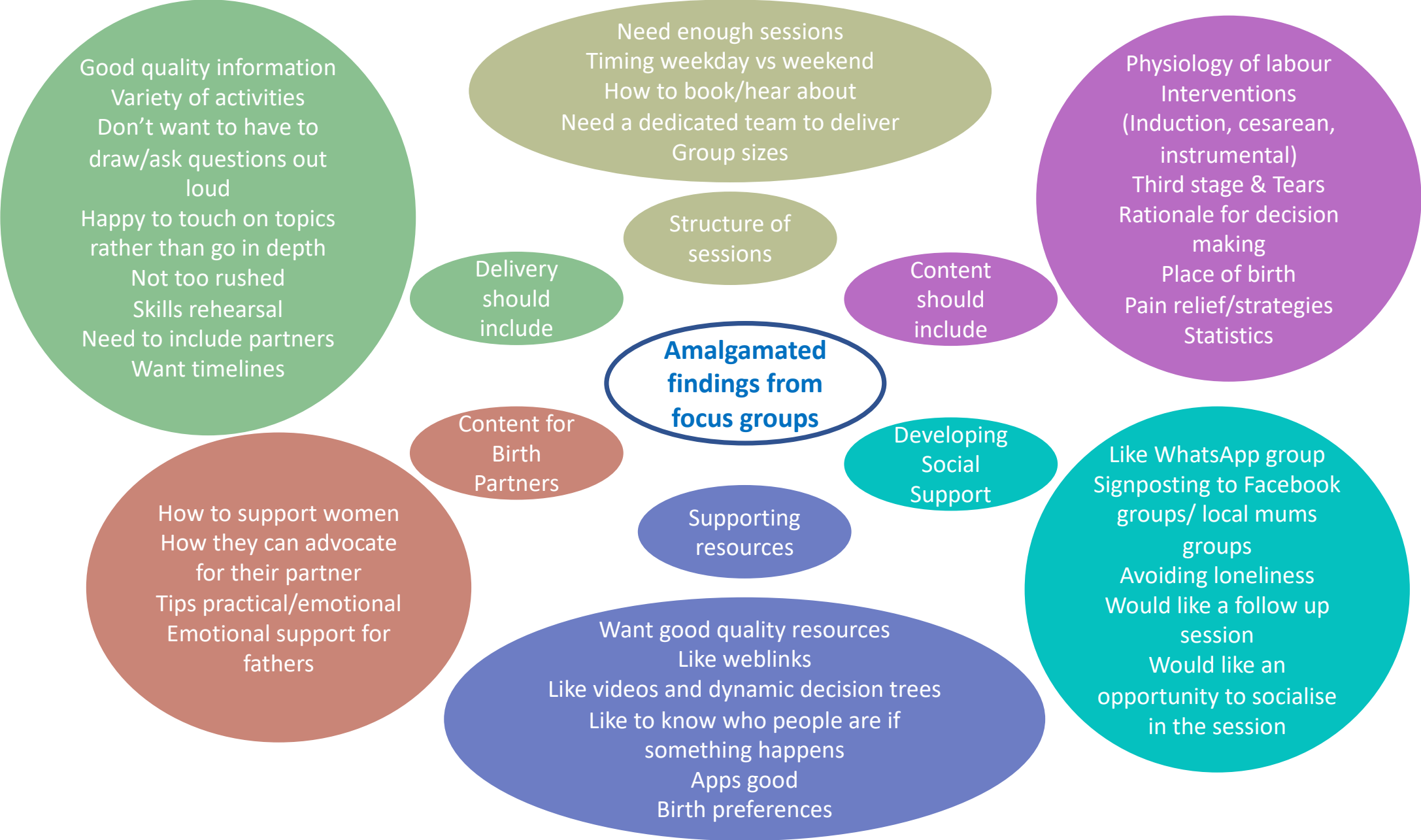

Supplement: Supplementary data [file bmjoq-2023-002731supp001.pdf]
