# Supplementary material for: Codesign and refinement of an optimised antenatal education session to better inform women and prepare them for labour and birth
Source: BMJ Open Qual. 2024 Jun 10;13(2):e002731. doi: 10.1136/bmjoq-2023-002731 (PMC11168157; doi:10.1136/bmjoq-2023-002731)
Supplement: Supplementary data [file bmjoq-2023-002731supp003.pdf]

Feedback from observed delivery of the 19 ACE sessions				
Topic covered	Yes	Partially	No	Not answered
**Section 1: Introducing the ACE programme and the river concept**	19/19			
**Section 2: Birth journeys**				
Topic 1: A straightforward vaginal delivery	19/19			
Topic 2: Assisted delivery with forceps and ventouse	19/19			
Topic 3: Caesarean Section deliveries	19/19			
Topic 4: Induction of labour journey	17/19	2/19		
**Section 3: Coping with labour and birth**				
Topic 1: Coping toolkit for labour and birth	16/19	2/19		1/19
Topic 2: Pain relief options (pharmacological)	19/19			
Topic 3: Non- pharmacological coping strategies	18/19	1/19		
Topic 4: partner support	17/19	1/19	1/19	
**Section 4:** Social support for the days and weeks following birth				
Topic 1: Caring for yourself after birth	14/19		1/19	4/19
Topic 2: Standard care	16/19	1/19		2/19
Topic 3: Support services	16/19	2/19		1/19
Topic 4: Social Connections	14/19	2/19		3/19
**Section 5**: Birth preferences	15/19	2/19		2/19
**Section 6:** Answering questions	16/19	1/19		2/19
**Section 7:** Social support opportunity	8/19		1/19	10/19
**Section 8:** Ending the session	9/19			10/19
**Use of resources**	15/19	2/19		2/19
**Use of what important to me**	2/19	3/19	12/19	2/19
**Videos used**	18/19			1/19
**Participants seemed engaged**	16/19	1/19		2/19
**Time**	8 sessions 120 +/- 5 mins<br>1 session 100 minutes<br>2 sessions 110-115 mins<br>3 sessions 40-47 mins over time (same facilitator)<br>5 session time not recorded			
**Additional Feedback from observers (general themes):**<br>Practical issues impacted some sessions (e.g. videos not working easily/late set up)<br>Timing: some classes over ran significantly<br>Classes were generally thorough, although occasionally some elements were missed<br>Questions were asked throughout and groups seemed to interact well				
