# Supplementary material for: Codesign and refinement of an optimised antenatal education session to better inform women and prepare them for labour and birth
Source: BMJ Open Qual. 2024 Jun 10;13(2):e002731. doi: 10.1136/bmjoq-2023-002731 (PMC11168157; doi:10.1136/bmjoq-2023-002731)
Supplement: Supplementary data [file bmjoq-2023-002731supp005.pdf]

Midwifery immediate feedback to researcher (general themes or important points raised by single person)	
Did the session go to plan	Question was answered in 12/19 sessions.<br>In 11/19 the feeling was yes but they altered the structure as required. 1/12 sessions they felt there wasn't enough time to impart all the information.
Things that went well	Facilitators liked the structure<br>They liked it when groups were interactive<br>They felt that women engaged<br>There were positive comments on group size, especially when the size was smaller.
Could go better	Want more time so sections less rushed<br>Practical issues – IT not working well and poster falling down<br>Would like to increase interaction
Suggested improvements to session	Need more time (possibility to split into more sessions)<br>Need to add in more about emergency situations<br>Improve the way the resources and the way they work (e.g. poster peeling off the wall, better sound for the videos, bigger posters without American spellings, and make posters more professional)<br>Suggestions about integration of the coping strategies into the birthing stages<br>The order felt obstetric, need more early labour coping strategies (1 participant)<br>Two people presenting would be better
Feedback from midwifery focus group	
General	Not enough time would like longer session<br>Equipment didn't work well at the start, others didn't have a problem with the IT<br>Paid closer attention to births other than normal (not usual for ANE classes)<br>Initially emergency situations not included enough this change was made and it was good to include as usually not addressed<br>Should include more postnatal information – others encouraged attendance at local infant feeding session<br>ACE resources make it more interactive<br>Not sure that the birth preferences tool worked well as redesigned for covid.
Resources	Journey a good resource<br>Video concept is good but lacks diversity<br>Having resources e.g. pelvis and ping pong balls, kiwi<br>Would have liked forceps
Manual and training	Used it for checking up, needs some elements e.g. emergencies adding to it<br>Coping strategies needs to be interspersed throughout<br>The training was fine, but need an hour to prepare and then session evolves over time
Social opportunity and how to improve	Didn't work well due to lack of interaction because of covid<br>Could introduce an interactive exercise for packing bag game<br>Could give out children's centre registration form
